# Viruses and Mitochondrial Dysfunction in Neurodegeneration and Cognition: An Evolutionary Perspective

**DOI:** 10.1007/s10571-024-01503-3

**Published:** 2024-10-17

**Authors:** George B. Stefano, Simon Weissenberger, Radek Ptacek, Martin Anders, Jiri Raboch, Pascal Büttiker

**Affiliations:** 1 Department of Psychiatry, First Faculty of Medicine, Charles University and General University Hospital in Prague, Ke Karlovu 11, 120 00 Prague, Czech Republic; 2 Department of Psychology, University of New York in Prague, Prague, Czech Republic

**Keywords:** Mitochondria, Virus, Bacteria, Cognition, Neurodegeneration

## Abstract

Mitochondria, the cellular powerhouses with bacterial evolutionary origins, play a pivotal role in maintaining neuronal function and cognitive health. Several viruses have developed sophisticated mechanisms to target and disrupt mitochondrial function which contribute to cognitive decline and neurodegeneration. The interplay between viruses and mitochondria might be traced to their co-evolutionary history with bacteria and may reflect ancient interactions that have shaped modern mitochondrial biology.

## The Evolution of Virus-Mitochondria Interactions

The relationship between viruses and mitochondria can be directly linked to their shared evolutionary history. Mitochondria originated from an ancient symbiosis between proto-eukaryotic cells and alphaproteobacteria that took place more than one billion years ago (Zimorski et al. 2019; Wang and Wu 2015; Vellai and Vida 1999). Mitochondria retain several features reminiscent of their bacterial origins, including numerous unique genomic DNA sequences and independent energy production machinery (Stefano et al. 2023; Wang and Wu 2015; Zimorski et al. 2019). This relationship suggests that co-evolving viruses, particularly bacteriophages, hereafter referred to as phages, may influence mitochondrial function as a vestige of these ancient relationships and/or via the acquisition of adaptations that enable them to exploit mitochondrial features.

Phages have driven the evolution of prokaryotic defense mechanisms over time (Pfeifer et al. 2022; Pfeifer and Rocha 2024). They facilitate horizontal gene transfer through transduction, which contributes to genetic variation and the spread of traits such as antibiotic resistance. Phages can replicate via the lytic cycle, in which they use the bacterial DNA replication machinery and are released into the environment upon lysis of the host cell. Alternatively, phages can enter the lysogenic cycle, in which they integrate themselves into the bacterial genome and remain dormant until conditions trigger lytic reactivation (Howard-Varona et al. 2017; Brussow et al. 2004).

**Fig. 1:**
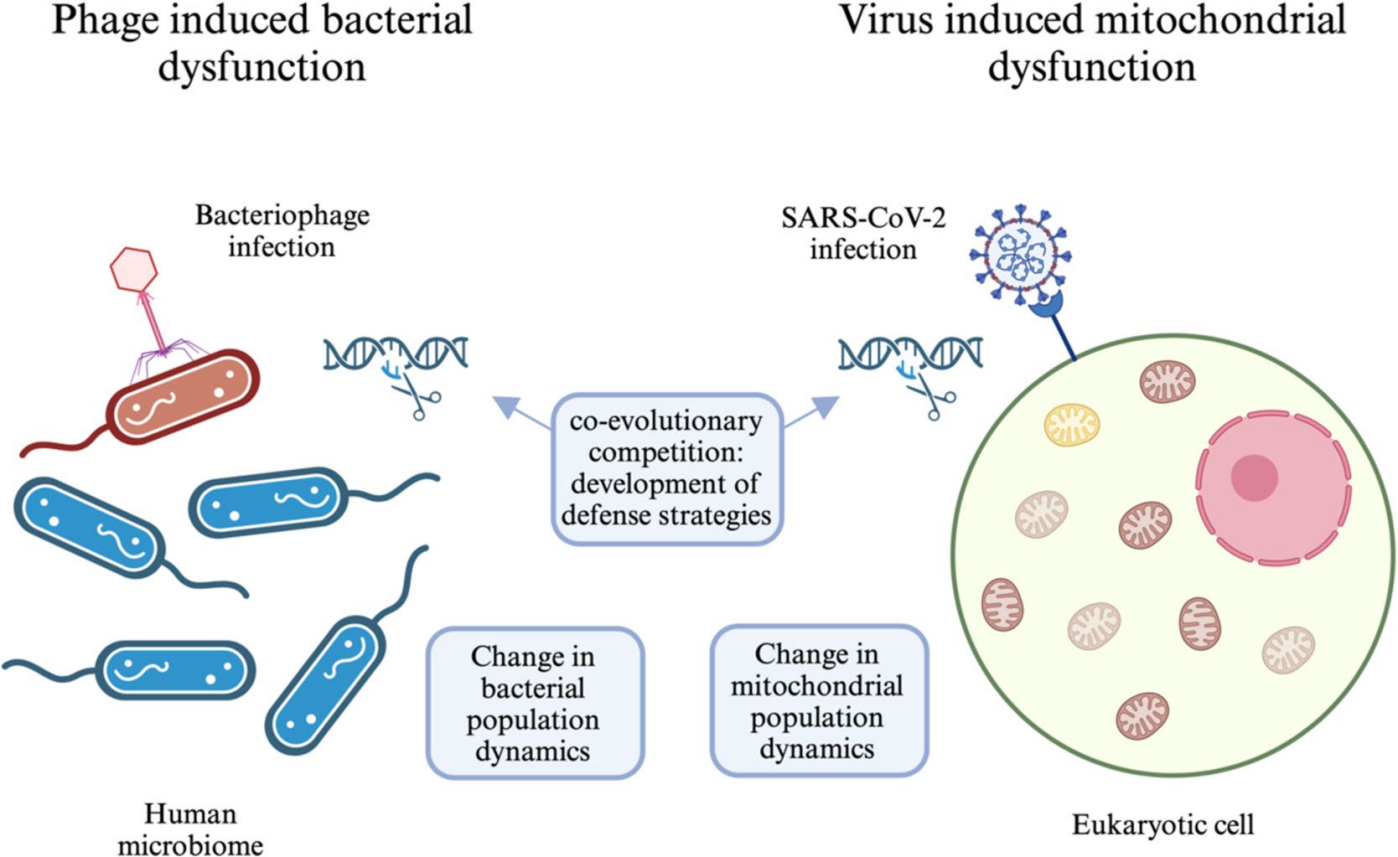
Viruses and bacteria represent co-evolutionary competition. These relationships drive bacterial evolution, e.g., as bacteria develop defenses, such as CRISPR-Cas systems, while phages evolve to overcome them, leading to a long successful relationship. Furthermore, given the bacterial origin of mitochondria, it is not surprising that this organelle is also a prime viral target, thus, continuing their relationship

Host-phage interactions may drive bacterial evolution (Fig. 1). Bacteria develop defenses, for example, clustered regularly interspaced short palindromic repeats and associated proteins (CRISPR-Cas). Phages then evolve to overcome these defense mechanisms, leading to a co-evolutionary competition (Sharma et al. 2023). Phages also regulate bacterial population dynamics in unique ecosystems, including the human microbiome, where they influence virulence through manipulation of the bacterial toxin-antitoxin system (Federici et al. 2021). Phages can also degrade biofilms by killing bacteria or alternatively, using them as platforms for infection (Sharma et al. 2023; Ranveer et al. 2024). Importantly, phages interact with their target host cells via mechanisms that are similar to those used by viruses that infect their eukaryotic counterparts (Huiting and Bondy-Denomy 2023). Similar to phages, viruses that infect eukaryotic cells are also capable of hijacking the DNA replication machinery, interrupting protein synthesis, and integrating themselves into host cell genomes, thereby affecting processes such as gene expression and cell division (Foo et al. 2022; Villion and Moineau 2013). Viruses can also interfere with metabolic pathways and divert energy production and nucleotide biosynthesis to support their own replication and exploit stress response pathways to induce dormancy, death, and other changes in their host ecosystems (Palmer 2022; Warwick-Dugdale et al. 2019). Collectively, these observations suggest that viruses that target eukaryotic cells and phages can alter the outcomes of biological pathways by redirecting key metabolic functions, driving evolutionary pressures, influencing virulence, and shaping ecological dynamics.

Given their bacterial origins, mitochondria may be uniquely vulnerable to viral manipulation. One example of this phenomenon is the virus-mediated disruption of mitochondrial apoptosis and energy production (Sorouri et al. 2022). These interactions could be remnants of ancient virus-bacteria dynamics or the result of evolutionary pressures that shaped viral strategies, permitting them to exploit several of the unique features of these organelles. Interestingly, recent evidence has revealed that mitochondria, similar to bacteria, are capable of releasing genetic material that subsequently integrates into the genomes of eukaryotic host cells, a phenomenon that can be considered analogous to virus insertional events, furthermore illustrating the evolutionary plasticity and universality of these systems (Stefano et al. 2023; Buttiker et al. 2022; Brandvain and Wade 2009; Bierne and Cossart 2012; Hu and Shu 2023). Collectively, these findings suggest that the molecular interactions among bacteria, viruses, and mitochondria, including the horizontal transfer of genetic material from the organelle to the eukaryotic genome, may be driven by one or more evolutionarily conserved mechanisms (Zhou et al. 2024).

This evolutionary perspective also provides a compelling framework for an understanding of how ancient virus-bacteria interactions continue to influence modern cellular biology. As discussed in the previous paragraph, viruses frequently target host mitochondria, disrupt energy metabolism, and modulate host protective behaviors to enhance viral infectivity and survival (Shin et al. 2024; Oh et al. 2024; Newman et al. 2024). This strategy not only supports immediate viral survival but also contributes to the long-term evolutionary success of these pathogens. For example, both human immunodeficiency virus (HIV) and cytomegalovirus (CMV) manipulate mitochondrial functions to evade host immune responses and establish persistent infections, thereby ensuring their longevity and propagation (Liu et al. 2019; Rasaiyaah et al. 2013). Additionally, the selective pressures exerted by viral infections on early eukaryotic cells may have driven the evolution of mitochondrial defenses and responses, further solidifying the virus-mitochondria relationship.

## Viral Disruption of Mitochondrial Function

Many viruses contribute to neurodegenerative processes by targeting mitochondrial function. For example, HIV-associated neurocognitive disorders are linked to the actions of the viral proteins Tat and gp120. These proteins disrupt mitochondria, leading to oxidative stress, DNA damage, and neuronal apoptosis (Lindl et al. 2010). Similarly, herpes simplex virus 1 (HSV-1) has been implicated in the pathogenesis of Alzheimer’s disease and can exacerbate oxidative stress, disrupt mitochondrial dynamics, and damage neurons (Feng et al. 2023). CMV also induces cognitive impairment, most notably in immunocompromised individuals, by generating reactive oxygen species (ROS) and damaging mitochondrial DNA (Combs et al. 2020). Zika virus also impairs mitochondrial function during fetal development, resulting in neuronal apoptosis and cognitive deficits, notably in cases of microcephaly (Komarasamy et al. 2022). Influenza virus has also been linked to cognitive deficits through mitochondrial dysfunction, including energy deficits and vulnerability to neurodegenerative processes (Jang et al. 2009; Marchi et al. 2023). Emerging evidence suggests that severe acute respiratory syndrome coronavirus 2 (SARS-CoV-2) similarly disrupts mitochondrial function via the actions of its spike protein and the resulting inflammatory response, thereby contributing to neuroinflammation and cognitive decline (Stefano et al. 2021, 2022; Davis et al. 2023). Collectively, these examples illustrate how many otherwise unrelated viruses target mitochondrial function, promoting neurodegeneration and cognitive impairment.

## How Did Viruses Shape Mitochondrial Defenses?

Viruses have had a profound influence on the evolution of mitochondrial responses and defense mechanisms. Over time, the competition between viruses and host cells has shaped mitochondrial functions in ways that enhance cellular defenses against viral infections. For example, mitochondrial antiviral signaling protein (MAVS) activates immune responses, which viruses can counteract via MAVS inhibition, thereby facilitating the evolution of enhanced host defenses (Chen et al. 2021). Similarly, viruses manipulate mitochondria-induced apoptosis to prolong the survival of infected host cells; this ultimately leads to the selection of cells that have developed more refined apoptotic mechanisms (Neumann et al. 2015). Likewise, although mitochondrial ROS production regulates immune responses to viral infections, viruses attempt to control and exploit this mechanism to support their own replication (To et al. 2020). Mitochondrial dynamics, including fission and fusion, have also been targeted by viruses to disrupt immune functions, while the release of mitochondrial DNA and autophagy (notably, mitophagy) serve as additional immune signals (Zong et al. 2024; Sorouri et al. 2022). Finally, viruses hijack mitochondrial metabolism to fuel their replication; in response, mitochondria have developed robust metabolic defenses against viral reprogramming. The ongoing evolutionary “battle” between viruses and mitochondria continues to shape both viral pathogenicity and host resilience.

## How Does This Communication Occur and Is So Effective?

The viral life cycle relies on the seamless integration of synthetic and packaging processes that take place within a host cell and are highly dependent on the precise pairing of complementary nucleotide sequences. The effectiveness of these processes may be crucially influenced by the functional outcomes of conformational matching or shape recognition events, which drive high-affinity binding interactions between complementary viral, host, and potentially bacterial nucleic acid and protein domains. Stereospecific conformational matching is a key feature of both intracellular and extracellular signaling pathways. This is somewhat predictable given that viruses, bacteria, and eukaryotic cells use the same nucleic acids which form the foundations of their interactions and life processes (Stefano 1986, 1988). Consequently, individual viral, host, and bacterial proteins are likely to exhibit some degree of similarity (e.g., molecular mimicry); thus, the existence of interactions between organisms remains unsurprising (Venigalla et al. 2020). This process can also conserve as well as modify information across phyla (English et al. 2023). Furthermore, this molecular mimicry between host and pathogen molecules arises, in part, due to the evolutionary conservation and temporal stability of host molecules. This stability provides a consistent structural target, facilitating the emergence of pathogen molecules that are stereospecifically complementary and conformationally matched. The rapid mutation rates of pathogens further enhance their ability to evolve such mimetic molecules, enabling them to exploit host molecular architecture effectively**.** Among these is the utility of retaining useful positive mutations that coordinate with the molecular shape recognition process. Conformational matching in signaling processes across diverse living organisms is subject to both micro- and macro-environmental influences that may alter the shape of the common biochemicals that are essential for their replication and survival. Thus, the thermodynamically driven suitability of these interactions is also a significant contributor to this process.

## Conclusion

Taken together, our findings suggest that the complex relationship between viruses and mitochondria is deeply rooted in their shared evolutionary history and that ancient virus-bacteria interactions have left a lasting imprint on modern cellular biology. Mitochondria, which have their origins in bacteria, remain critical targets for the many viruses capable of exploiting this ancient connection to disrupt mitochondrial function. These interactions ultimately lead to cognitive decline and neurodegeneration. The diverse mechanisms used by modern viruses such as HIV, HSV, CMV, Zika virus, Influenza, and SARS-CoV-2 highlight the pivotal role played by mitochondrial dysfunction in viral pathogenesis and the broader implications for neuronal health. Understanding these evolutionary dynamics not only provides insight into the mechanisms of neurodegenerative diseases but also underscores the importance of mitochondria as a focal point in the ongoing conflict between host defense systems and viral survival strategies. This knowledge may pave the way for novel therapeutic approaches aimed at preserving mitochondrial function to combat virus-induced cognitive impairment and neurodegenerative disorders.

## Data Availability

No datasets were generated or analysed during the current study.
